# Supplementary material for: Exploiting induced pluripotent stem cell-derived macrophages to unravel host factors influencing Chlamydia trachomatis pathogenesis
Source: Nat Commun. 2017 Apr 25;8:15013. doi: 10.1038/ncomms15013 (PMC5414054; doi:10.1038/ncomms15013)
Supplement: Supplementary Information — Supplementary Figure and Supplementary Table [file ncomms15013-s1.pdf]

Supplementary Figure 1: Validation of the mutants by sequencing and phenotypic assays. Panels A and B showed the bi-allelic mutations were validated by PCR and sequencing. Specifically, the insertion of the cassette into the correct locus was confirmed by visualising on 1% E-gel (Life Tech) PCR products of indicated sizes were generated by gene specific (GF1 and GR1) and cassette specific (ER and PF) primers for both 5' and 3' ends (Supplementary Data 1 and Panel A). To check the CRISPR site on the non-targeted allele, PCR products were generated either from across the locus, using the 5' and the 3' gene specific genotyping primers (GF1-GR1), or from around the site using primers 5F-3R (Supplementary Data 1) that would amplify a short, around 500 bp, amplicon. In both cases the PCR products were treated with exonuclease and alkaline phosphatase (NEB) and Sanger sequenced using primers SF and SR (Supplementary Data 1 and Panel B). Both mutant human iPS lines were able to differentiate into macrophages and expressed macrophage markers (CD11b, CD14, CD16, CD68 as measured by flow cytometry; red histograms represent cells stained with control isotype and blue histograms represent cells stained with relevant antibody; Panel C). Both mutant iPSdMs showed an ability to phagocytose using pHrodo Green dye-labeled E.coli (ThermoFisher) and measuring increased fluorescence by flow cytometry- red histograms represent untreated cells and blue histograms represent cells treated with pHrodo Green dye labelled E.coli for 30 mins (Panel D). For clarity we only show representative flow cytometry plots for the IRF5$^{-/-}$ H09 and IL10RA$^{-/-}$ D07 mutant clones but we observed similar macrophage markers and phagocytic results for the IRF5$^{-/-}$ F07 and IL10RA$^{-/-}$ E09 mutant clones.

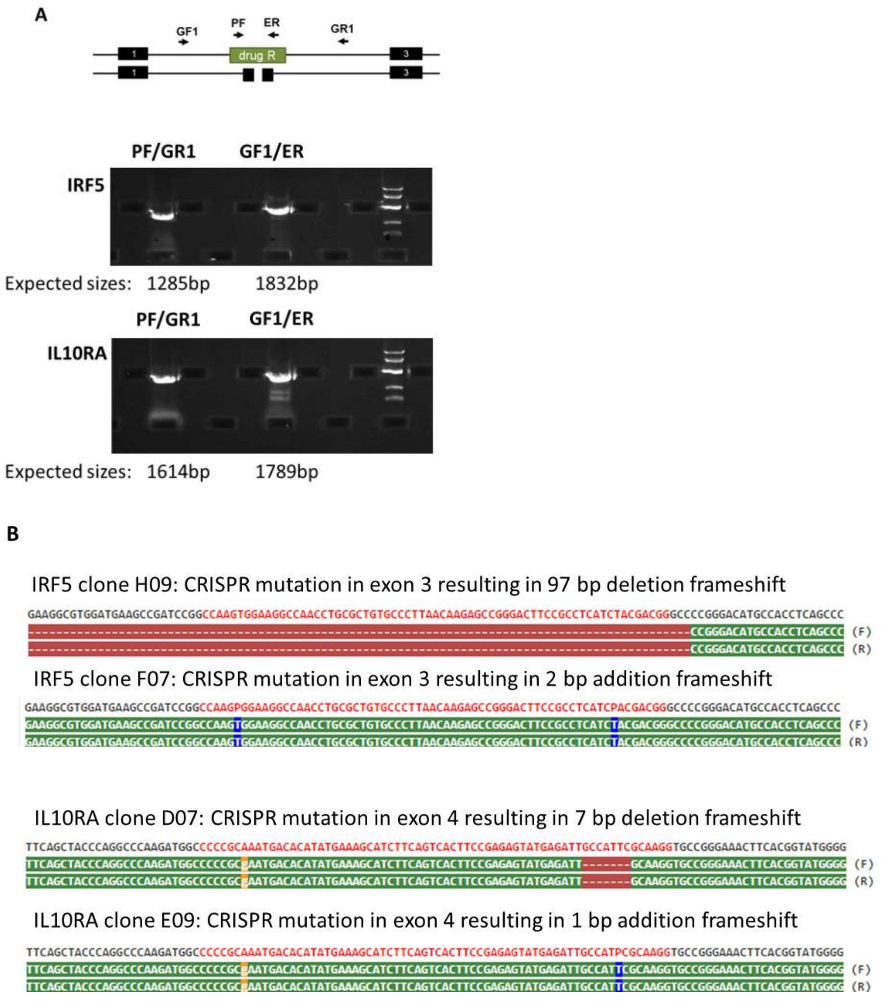

C

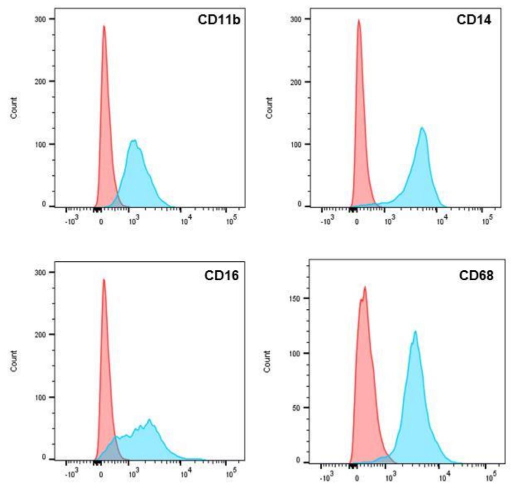

D

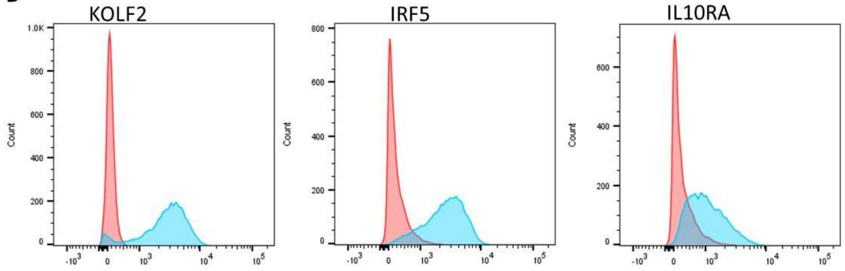

Supplementary Table 1: PCR primers and CRISPR sequences utilized in generation and genotyping of IRF5-/- and IL10RA-/- iPSC mutant clones.

**CRISPR sites (PAM site underlined)**	
Left Crispr_IRF5	CCAAGTGGAAGGCCAACCTGCGC
Right Crispr_IRF5	GACTTCCGCCTCATCTACGACGG
Left Crispr_IL10RA	CCCCGCAAATGACACATATGAAA
Right Crispr_IL10RA	GTATGAGATTGCCATTCGCAAGG
**Gibson primers (append sequence underlined)**	
IRF5_5F	AACGACGGCCAGTGAATTCGATGTCATGTAGGCTG TCTTAATGCTTC
IRF5_5R	TATCGTTATGCGCCTTGATCTGTCCCATAGGAGACT CTACTGT
IRF5_3F	CTGAGCTAGCCATCAGTGATGATGGGTCACTGGCA TATCAGGAAT
IRF5_3R	CCATGATTACGCCAAGCTTGATAGTAAAGAATAGG GTGTCATGTGGG
	_
IL10RA_5F	AACGACGGCCAGTGAATTCGATTATTTCGCTCATG ATGTCCTCTAGG
IL10RA_5R	TATCGTTATGCGCCTTGATGAATACCATGAGACTT GAGGGCTG
IL10RA_3F	CTGAGCTAGCCATCAGTGATGTGCCATTGGGAACT TTGCTTAT
IL10RA_3R	CCATGATTACGCCAAGCTTGATTGAAATTTGTTGC AGATCTAGCAGG
**Universal sequencing primers, Intermediate vector**	
p19F	aactgttgggaagggcgatc
ZP1	GAAGTCGTCCTCCACGAAGT
ZP2	GAACTGTGGTTACGCGAATG
p19R	gttagctcactcattaggcac
**Universal sequencing primers, Final vector**	
p19F	aactgttgggaagggcgatc
EF1aR1	CTCTGGGTTCTACGTTAGTG
pA_R	CGCGTCGAGAAGTTCCTATTC
p19R	gttagctcactcattaggcac
**Cassette PCR primers for genotyping targeted allele**	
ER	TGATATCGTGGTATCGTTATGCGCCT
PF	CATGTCTGGATCCGGGGGTACCGCGTCGAG
**Gene specific PCR primers for**	

**genotyping targeted allele**	
IRF5_GF1	GACTGTAGAATTTCCACTGGAATGG
IRF5_GR1	CGGCCACTTGACATCCTCTTTG
IL10RA_GF1	TATGGCATTTATCTCTTTGCACCTG
IL10RA_GR2	TCCTCTGCATTGAGACTTAAATGAC
**Gene-specific PCR primers for genotyping non-target allele**	
IRF5_PR2	GTAAAGGAGTCAATGTCGGAGGAG
IRF5_PF1	CAACTGTGAGGAGATCAAGACAAAG
IL10RA_PF1	TATTTCGCTCATGATGTCCTCTAGG
IL10RA_PR1	GAACAGCAGAAGACTGTAACAACAG
**Gene-specific sequencing primers for genotyping non-targeted allele**	
IRF5_SR1	GTGTCATGACCTAGCGGGAG
IRF5_SF1	GGAGCAGGGACTATGGATGC
IL10RA_SF1	TGACAAACCTGTGGCCAAGT
IL10RA_SR1	TTCTACTCCCTCCTCTGCCC
